# Regional disparities of full pentavalent vaccine uptake and the determinants in Ethiopia: Mapping and spatial analysis using the EDHS data

**DOI:** 10.1371/journal.pone.0312514

**Published:** 2025-01-09

**Authors:** Getasew Mulat Bantie, Melaku Tadege, Teshager Zerihun Nigussie, Ashenafi Abate Woya, Abay Kassa Tekile, Amare Alemu Melese, Simeneh Ayalew, Belay Bezabih Beyene, Gizachew Yismaw Wubetu

**Affiliations:** 1 Regional Data Management Center for Health, Amhara National Regional State Public Health Institute, Bahir Dar, Ethiopia; 2 Department of Statistics, College of Science, Debre Tabor University, Debre Tabor, Ethiopia; 3 Department of Statistics, College of Science, Bahir Dar University, Bahir Dar, Ethiopia; 4 Ethiopian Public Health Institute, Addis Ababa, Ethiopia; 5 Amhara National Regional State Public Health Institute, Bahir Dar, Ethiopia; King Faisal University, SAUDI ARABIA

## Abstract

**Background:**

The full pentavalent (DPT-HepB-Hib) vaccination is the main strategy to prevent five communicable diseases in early childhood, especially in countries with huge communicable disease burdens like Ethiopia. Exploring spatial distributions and determinants of full pentavalent vaccination status in minor ecological areas in Ethiopia is crucial for creating targeted immunization campaigns and monitoring the advancement of accomplishing sustainable development goals. This study aimed to investigate the spatial disparities and determinants of full pentavalent vaccination among 12-23-month-old children in Ethiopia.

**Method:**

The data on pentavalent vaccine uptake was found in the Ethiopian Health and Demographic Survey (EDHS, 2019). A two-stage cluster sampling method was applied to collect the EDHS data. The enumeration area was the primary sample unit while the household served as the secondary sampling unit. The geographical variations of full pentavalent vaccine uptake were explored using Quantum Geographic Information System (QGIS) software. The significant predictors of full pentavalent vaccination were identified using a simple logistic regression model through R version 4.1 software.

**Result:**

The national full pentavalent vaccine uptake was 59.2%. The spatial distribution of full pentavalent vaccine uptake was not uniform in Ethiopia. Spatial cluster analysis revealed that most of low coverage regions for full pentavalent vaccine uptake were Afar, Somali, and Harari. The regions with the highest and lowest rates of vaccine uptake were Tigray and Harari region, respectively. Maternal age of 35–49 years (AOR = 3.42; 95% CI: 1.99, 5.87), and 25–34 years (AOR = 1.55; 95% CI: 1.17, 2.19), primary education attended (AOR = 1.51; 95%CI: 1.07, 2.11), richness wealth index (AOR = 1.96; 95% CI: 1.40, 2.75), birth order of 1–3 (AOR = 1.88; 95% CI: 1.19, 2.96), and delivery in the health facility (AOR = 3.41: 95% CI: 2.52, 4.61) were the determinants of full pentavalent vaccine uptake in Ethiopia.

**Conclusion:**

Ethiopia’s full pentavalent vaccine uptake was far lower than the global target. Older maternal age, maternal education, wealth index, birth order, and giving birth in a health facility were the determinants of full pentavalent vaccine uptake. Special attention should be given to Afar, Somali, and Harari regions, to strengthen the vaccine uptake. Moreover, improved socioeconomic status and getting maternal health services during delivery are necessary to enhance vaccine uptake.

## Background

Childhood vaccination is a global cost-effective health intervention and a mainstay for maintaining Universal Health Coverage (UHC) [1]. Combining vaccines to administer in a single syringe has paramount importance for controlling vaccine-preventable diseases [2]. Pentavalent vaccine contains five antigens administered in a single dose to protect five different illnesses: diphtheria, pertussis (whooping cough), tetanus, hepatitis B, and Haemophilus influenza B [3]. Moreover, it reduces clinic visits, logistical challenges, trauma, higher vaccination compliance, and allows the incorporation of new vaccines into immunization schedules [2, 4–11].

Ethiopia began administering the pentavalent vaccination as part of its national immunization program in 2007, as part of a global initiative [12]. Since then, Ethiopia has implemented a number of plans to increase the country’s pentavalent vaccination coverage by combining the Reaching Every District method, the health extension program, and the Enhanced Routine Immunization Activities [13–15].

Nevertheless, despite all of these outstanding efforts, the country’s level of full pentavalent vaccine coverage is still low [16]. Various studies revealed that socio-demographic, economic, and healthcare access-related characteristics have an impact on the coverage of full vaccination [17–24]. All these studies did not consider geographic variation in the estimation of full vaccine uptake. Exploring geographic disparities and inequalities of full pentavalent vaccination coverage in small geographical areas is critically important for designing targeted immunization campaigns and allocating equitable resources.

Despite few geospatial studies in vaccination related issues are available, no study has shown the full pentavalent vaccine uptake in recent years in Ethiopia. Hence, this study was designed to investigate the spatial disparities and the determinants of full pentavalent vaccination among 12–23 months children in Ethiopia.

## Methods

### Study design, area, and data source

This cross-sectional study was done using the 2019 Ethiopian demographic and health survey dataset. Ethiopia is a landlocked East African country located 30–140 N and 330-480E with 1.1 million Square. kilometer coverage [25]. The survey covered the Ethiopian nine regions and two city administrations [26].

### Data collection tools and procedures

A two-stage stratified cluster sampling were employed to collect the data and encompassed 305 enumeration areas. The enumeration areas were the primary sampling units. From each respective cluster, 30 households were randomly selected. The households were the secondary sampling units. Finally, 1,008 mothers with children 12 and 23 months of age were interviewed about their children pentavalent vaccination status within five years preceding the 2019 EDHS were included [26]. The WHO and DHS program including the Ethiopian ministry of health uses children age category between 12–23 months as a reference for full vaccine uptake assessment [27].

### Immunization program in Ethiopia

Ethiopia introduced the pentavalent vaccine in the national routine immunization program in 2007 [12]. Since then, to enhance the national pentavalent vaccine uptake, the Ethiopia government has been implementing several strategies; like free of charge of the services and reaching every district via static, outreach and mobile approaches [13–15]. Immunization services are being delivered at all levels of healthcare delivery system [28] and national vaccination schedule are shown below [29] (Table 1).

**Table 1:** Ethiopian national routine vaccination schedule, 2022.

Antigen	Total Doses	Recommended Age	Route/Site of Administration
HepB Vaccine Birth dose	1	At birth or within 24 hours of birth. For home delivered baby, vaccinate up to 14 days old.	Intramuscular IM, Lt anterolateral thigh
BCG	1	At birth or soon after	Intradermal (ID), Rt deltoid
Polio (OPV)	4	Birth (OPV0), weeks 6, 10, & 14	Oral
DPT-Hib-HepB (Pentavalent)	3	Weeks 6, 10, & 14	IM, Lt anterolateral thigh
PCV	3	Weeks 6, 10, & 14	IM, Rt anterolateral thigh
Rotavirus vaccine	2	Weeks 6 & 10	Oral
IPV	14	Week 14	IM, Rt thigh 2.5 cm below PCV injection site
MCV	2	9 and 15 months	Subcutaneous (SC), Rt deltoid

### Data measurement

Full pentavalent vaccine uptake is defined as a child that has received three doses of pentavalent vaccines [30]. The outcome variable for the study was full pentavalent vaccine uptake. Maternal age, maternal educational status, residence, household income, mother currently pregnant, birth order, place of delivery, and delivery by caesarean section were the explanatory variables.

The data on vaccination coverage in the 2019 mini-EDHS report was gathered from immunization cards presented to the interviewers as well as from mothers’ verbal accounts. When immunization cards were accessible, the interviewer transcribed the vaccination dates directly onto the questionnaires. If the child did not have a vaccination card or if a vaccine was not documented on the card, respondents were asked to recall whether their child had received the vaccine.

The authors categorized full pentavalent vaccination as "0" for children who did not receive all three doses of the pentavalent vaccines, labeling them as ’full pentavalent vaccine not uptake.’ Conversely, they assigned a "1" to children who received all three doses, classifying them as ’full pentavalent vaccine uptake.’ This classification was based on maternal reports and the information recorded on the child’s vaccination card.

### Data analysis and management

Data analysis was done using R and QGIS software. The full pentavalent vaccine uptake for each of the selected EAs and regions (with 95% confidence interval) was calculated by taking into account the complex survey sampling design.

### Geospatial data processing

The full pentavalent vaccination dataset was arranged in spatial form using a hierarchical logistic regression model. A spatial model was used to discern spatial patterns of full pentavalent vaccine uptake and to explore different spatial covariance structures. The study area’s overweight patterns were measured using a spatial autocorrelation (Global Moran’s I) statistic. A Moran’s I that was statistically significant (p < 0.05) was employed to measure spatial autocorrelation. Additionally, the overweight of the nation’s unsampled areas was predicted using a spatial interpolation technique based on sampled EAs and Ordinary Kriging spatial interpolation methods.

### Ethics approval and consent to participate

The data used for this study are aggregated secondary data that are publicly available with no personal identifiers that are directly allied to the study participants. However, a formal request was sent to DHS website by filling in the online request form.

## Results

### Sociodemographic and obstetric characteristics of the study participants

In this study 1008 study participants took part. The mean (±SD) age of the respondents was 28.6 (±6.5) years. More than half (52.8%) of the respondents were in the age groups of 25–34 years. About three-forth (73.5%) of the respondents were rural residents. Nearly half (48.9%) and 46% of the respondents had not attended formal education and were in the poor/poorest wealth status. About forty three percent of the participants gave birth in their home (Table 2).

**Table 2:** Socio-demographic characteristics of the respondents, in Ethiopia, 2019.

Variable	Category	Frequency	Percent
Maternal age	15–24 years	298	29.6
	25–34 Years	532	52.7
	35–49 years	178	17.7
Place of residence	Urban	267	26.5
	Rural	741	73.5
Maternal education	No education	493	48.9
	Primary	342	33.9
	Secondary	99	9.8
	Higher	74	7.4
Wealth index	Richer/richest	403	40.0
	Middle	136	13.5
	Poorer/poorest	469	46.5
Mother currently pregnant	No or unsure	901	89.4
	Yes	107	10.6
Birth order	1–3	602	59.7
	4–5	204	20.2
	6 and more	202	20.1
Delivery place	Health Institution	577	57.2
	Home	431	42.8
Delivery by caesarean section	Yes	71	7.0
	No	937	93.0

### Full pentavalent vaccine uptake

**Fig 1:**
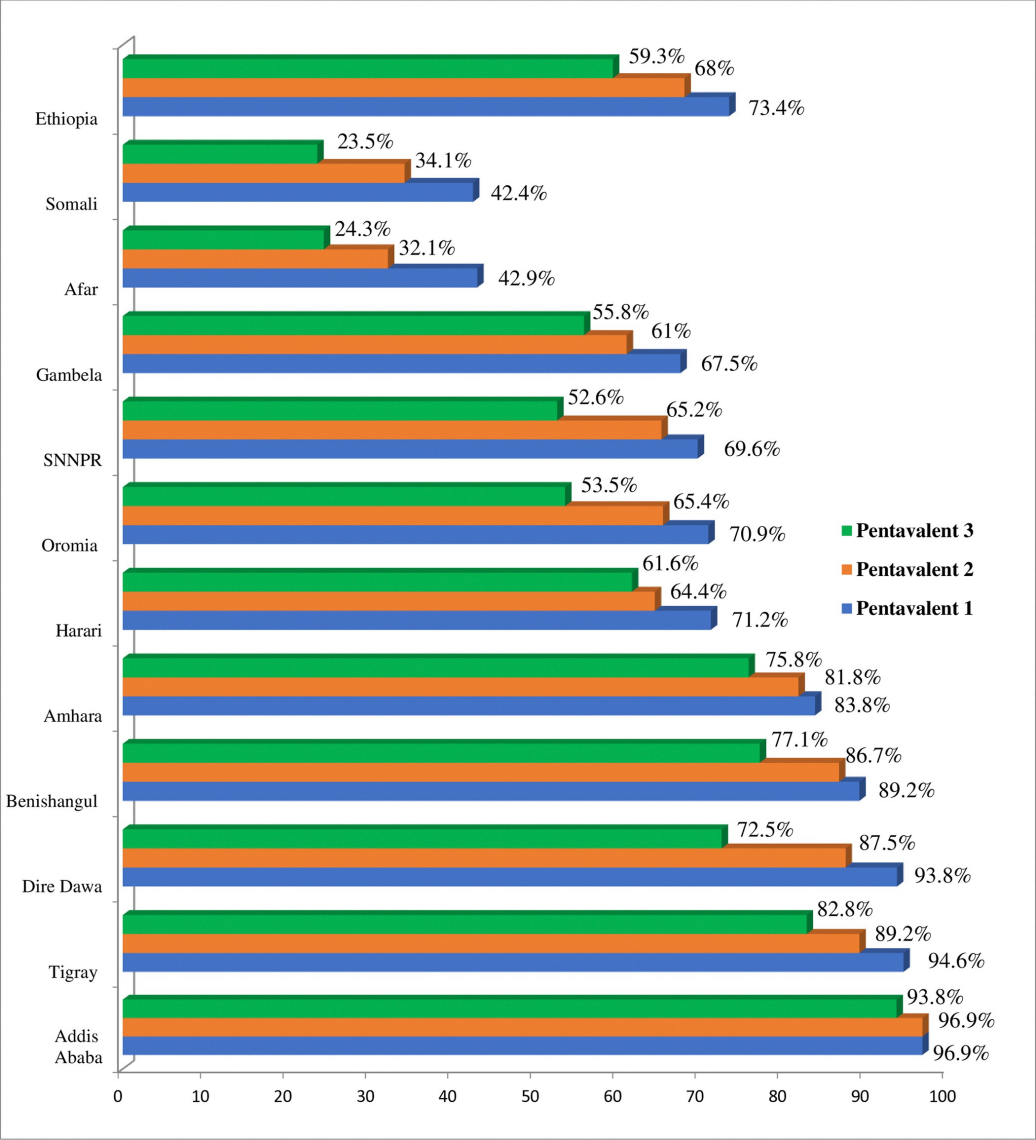
Regional full pentavalent immunization uptake disparities in Ethiopia, 2019.

The full pentavalent vaccine uptake (pentavalent 3) in 2019 was 59.2% (95%CI: 56, 62) with significant variances among regions. The Somali area had the lowest full pentavalent vaccine uptake (23.5%), whereas Addis Ababa city had the highest (93.68%) uptake (**Fig 1**).

### Spatial analysis of full pentavalent vaccine uptake

**Fig 2:**
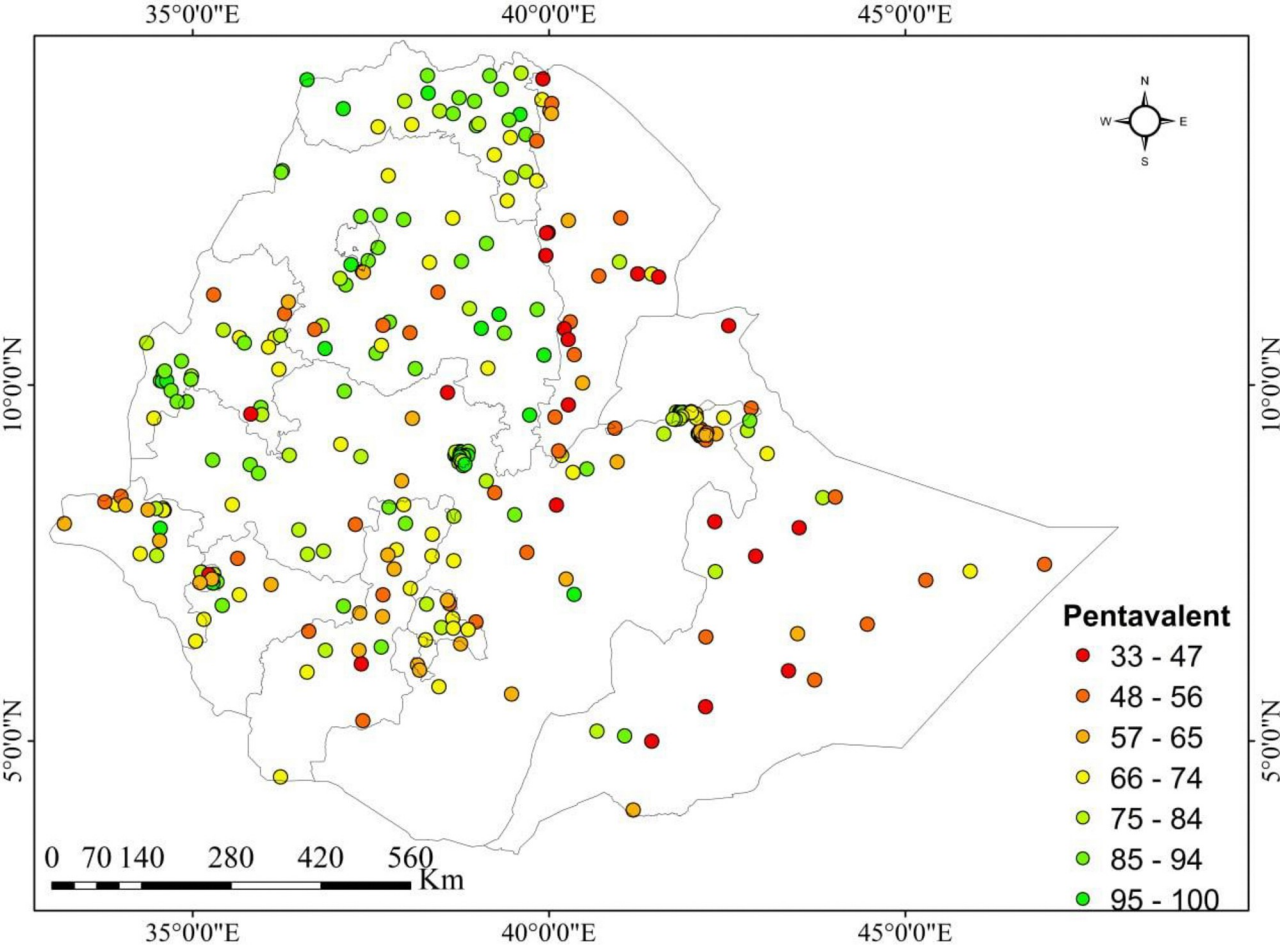
Geographical depiction of full pentavalent immunization uptake in Ethiopia, 2019.

Each point in the map characterizes one enumeration area with the proportion of full pentavalent vaccine uptake in each cluster. The regions with the highest rates of vaccine uptake were Tigray, Dire Dawa, Gambela, Addis Ababa, and the Northeastern section of the South Nation Nationalities and Peoples Region (SNNPR). On the other hand, Harari region, East Afar, the border between Benishangul Gumuz and Oromia, the central portion of Somalia, the border between Amhara and Benishangul Gumuz, and the east of Oromia had the lowest rates of full pentavalent vaccine uptake (**Fig 2**).

**Fig 3:**
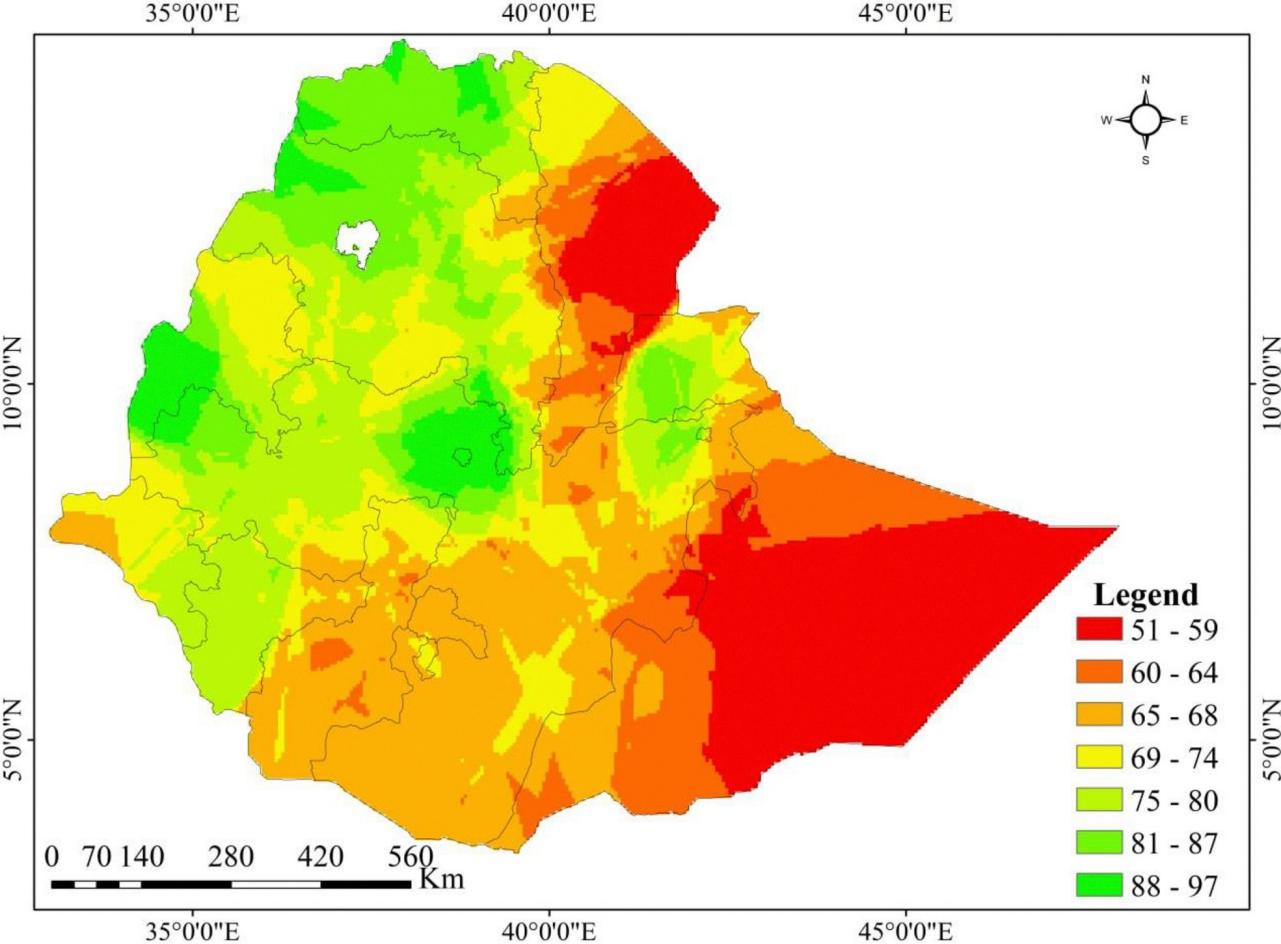
Kriging interpolation of full pentavalent immunization among children aged between 12 to 23 months in Ethiopia, 2019.

Based on measurements of the sampled enumeration area, the spatial kriging interpolation approach was utilized to forecast full pentavalent vaccine uptake in unsampled regions of the nation. Red forecasts denoted areas with the lowest vaccine uptake rates. When compared to other regions, it was anticipated that vaccine uptake would be lowest in Afar, Somali, Harari, Southwest SNNPR, East Benishangul Gumuz, and the South and Central area of Oromia. On the other hand, it was anticipated that the following regions will have significant (higher) vaccine uptake: Tigray, Amhara, Addis Ababa, Northwest Oromia, North Somali, Dire Dawa, Northwest Benishangul Gumuz, Northeast SNNPR, border of Gambela, and SNNPR, as well as border of Gambela and Oromia (**Fig 3**).

### Spatial autocorrelation analysis

**Fig 4:**
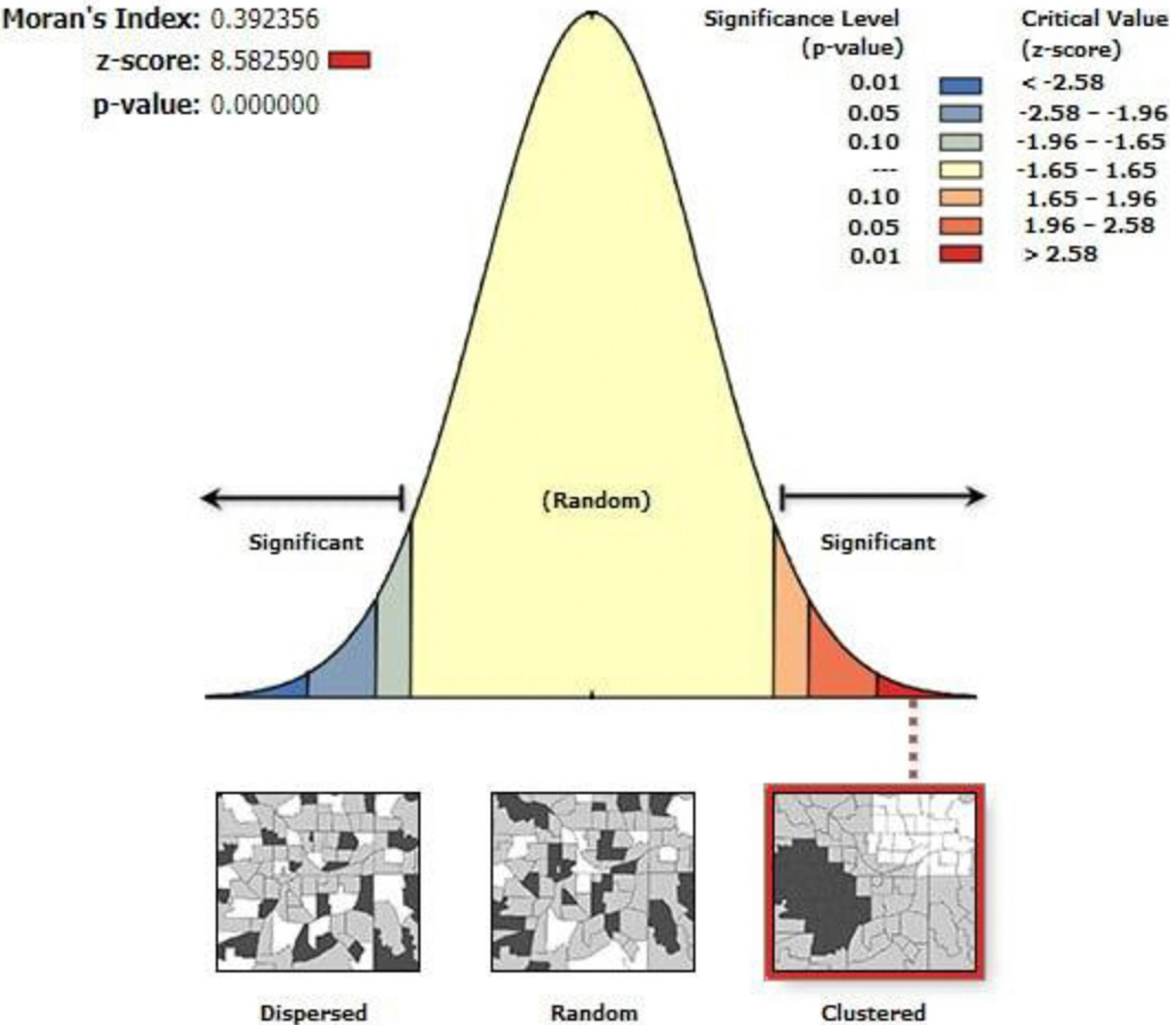
The overweight patterns of autocorrelation of full pentavalent immunization uptake in Ethiopia, 2019.

Based on the current study finding, the distribution of full pentavalent vaccine uptake was significantly clustered in Ethiopia (**Fig 4**).

### Determinants of full pentavalent vaccine uptake

In the model; maternal age, educational status, place of delivery, birth order, and the wealth index were the determinants for full pentavalent vaccine uptake.

For mothers whose age was 35–49 years and 25–34 years, the likelihood of the children uptaking the full pentavalent vaccine uptake were about 3.5 (AOR = 3.42, 95% CI: 1.99, 5.87) and 1.6 (AOR = 1.55; 95%CI:1.17, 2.19) times higher than those of mothers whose age 15–24 years.

Similarly, mothers who were attending primary education, the likelihood of the children receiving full pentavalent vaccination were nearly 1.5 (AOR = 1.51; 95%CI: 1.07, 2.11) times higher than the counterparts. For children who belonged to the wealthy family, the chance of receiving full pentavalent vaccination were nearly twofold (AOR = 1.96; 95%CI: 1.40, 2.75) higher than those of children from poverty-stricken families.

In children whose birth order was between one and three, the odds of receiving full pentavalent vaccination were about twofold (AOR = 1.88; 95%CI: 1.19, 2.96) higher compared to children whose birth order was six or more. Similarly, the odds of receiving full pentavalent vaccination among children who were delivered in a health institution was 3.4 (AOR = 3.41; 95%CI: 2.52, 4.61) times higher that delivered at home **(Table 3).**

**Table 3:** Binary logistic regression analysis to identify determinants of full pentavalent vaccination coverage among 12–23-month children in Ethiopia, 2019.

Variable	Category	Full Pentavalent Vaccination		COR (95%CI)	AOR (95%CI)	P-value
		No	Yes			
Maternal age	15–24 years ^*R*^	138	160	1.00	1.00	0.013
	25–34 Years	215	317	1.27 (0.96, 1.69)	1.55 (1.17, 2.19)	
	35–49 years	58	120	1.78 (1,21, 2.63)	3.42 (1.99, 5.87)	0.0001
Place of residence	Urban	58	209	3.28 (2.37, 4.54)	1.36 (0.89, 2.06)	0.157
	Rural ^*R*^	353	388	1.00	1.00	
Maternal education	No education ^*R*^	249	244	1.00	1.00	0.019
	Primary	121	221	1.86 (1.41, 2.47)	1.51 (1.07, 2.11)	
	Secondary	27	72	2.72 (1.69, 4.38)	1.44 (0.82, 2.51)	0.202
	Higher	14	60	4.37 (2.38, 8.03)	1.48 (0.74, 2.96)	0.273
Wealth index	Richer/richest	98	305	3.71 (2.77, 4.96)	1.96 (1.40, 2.75)	0.0001
	Middle	58	78	1.61 (1.09, 2.36)	1.16 (0.77, 1.76)	0.47
	Poorer/poorest ^*R*^	255	214	1.00	1.00	
Mother currently pregnant	No or unsure	356	545	1.62 (1.08, 2.42)	1.27 (0.81, 1.98)	0.301
	Yes	55	52	1.00	1.00	
Birth order	1–3	224	378	1.53 (1.11, 2.11)	1.88 (1.19, 2.96)	0.007
	4–5	91	113	1.13 (0.76, 1.66)	1.53 (0.97, 2.40)	0.068
	6 and more ^*R*^	96	106	1.00	1.00	
Delivery place	Health Institution	148	429	4.54 (3.47, 5.94)	3.41 (2.52, 4.61)	0.0001
	Home ^*R*^	263	168	1.00	1.00	
Delivery by caesarean section	Yes	17	54	2.31 (1.32, 4.04)	0.76 (0.41, 1.40)	0.379
	No ^*R*^	394	543	1.00	1.00	

R = reference category: COR = crude odds ratio, AOR = adjusted odds ratio, CI = confidence interval

## Discussion

Vaccination is an essential part of primary health care and is one of the most effective health investments available. It plays a crucial role in preventing and managing infectious disease outbreaks [31]. Hence, this study aimed to assess the regional disparities of full pentavalent uptake and the determinants in Ethiopia and will help to fill the gaps/variations observed in vaccine uptake across regions of the country [32]. The current study revealed that the overall full pentavalent vaccine uptake in Ethiopia was 59.2% (95%CI: 56% - 62%). This finding is consistent with the study findings of the EDHS-2016, 56.1%, southern Benin 57.14%, and Nigeria 58% [23, 33, 34].

However, the current study is much lower than the study findings of Sekota Zuria district 77.3% [35], Minjar Shenkora district, Ethiopia 92.8% [22], Gondar town, Ethiopia 75.5% [36], Woldiya, Ethiopia 87.7% [37], Worabe town Ethiopia 87.4% [38], Kenya 90% [39], East Africa 69.21% [40], Togo 63.7% [41], Sierra Leone 86.3% [42], Senegal 82.6% [43], Benin 85.5% [44], Ghana 89.5% [45], and southern India 96% [46].

In contrast, the current study finding was bigger than the findings of Dabat demographic and health survey site 30.9% [47], Afar 20.6% [48], Somali 27.5% [49], Mizan Aman town 42.2% [50], Oromia 25% [51], Haremaya District, Eastern Ethiopia 50.6% [52], Uganda 52.5% [53], Cameroon 53.6% [54], Coted’Ivoire 50.5% [55], DR Congo 49.8% [56], Haiti 45.8% [57], and Pakistan 58.47% [58]. This variation might be due to some of the former studies were carried out in nomadic and pastoralist communities in the Afar and Somali regions, where weak healthcare systems hinder immunization. Moreover, differences in health systems and immunization policies, varying awareness of vaccination services, and socio-cultural factors across countries also might contribute for the difference. Additionally, disparities in the availability and accessibility of immunization services may contribute to vaccine hesitancy, influenced by cultural misconceptions and concerns about adverse effects [23, 36, 40, 52].

This study revealed that the full pentavalent vaccination coverage in Ethiopia varied greatly among administrative regions, ranging from 23.5% in Somali to 93.8% in Addis Ababa. Spatial autocorrelation analysis also showed that full pentavalent vaccine uptake in Ethiopia was not randomly distributed, with a global Moran’s I value of 0.3923 (p < 0.0001), indicating significant clustering across regions. The regions with the highest rates of vaccine uptake were clustered in Tigray, Dire Dawa, Gambela, Addis Ababa, and the Northeastern section of the South Nation Nationalities and Peoples Region (SNNPR). On the other hand, Harari region, East Afar, the border between Benishangul Gumuz and Oromia, the central portion of Somalia, the border between Amhara and Benishangul Gumuz, and the east of Oromia had the lowest rates of full pentavalent vaccine uptake. This was in agreement with the other indigenous study findings [59, 60]. The possible reasons might be sociodemographic (like mothers’ age, formal education, wealth status, and residence), differences in health service availability, access, counselling, awareness of vaccination schedules, and health-seeking behavior among regions [61–63]. In addition, most regions with low full pentavalent vaccination coverage had weak healthcare systems, which led to low vaccine uptake. Besides, a few sub-nations like Afar and Somali had hard-to-reach nomad and pastoralist tenants with no lasting home [64]. Hence, the current full pentavalent vaccine uptake finding reported as it is not promising for achieving a 2025 health sector transformation plan of 90% [51].

Maternal characteristics, age, educational level, wealth index, order of birth, and place of birth attended were the determinants of full pentavalent vaccine uptake.

For mothers whose age was 25–49 years, the likelihood of the children to uptake the full pentavalent vaccination was higher than those of mothers whose age 15–24 years. This could be attributed to older mothers having more experience and a better understanding of the significance and impact of immunization compared to younger mothers. This finding is consistent with research conducted in Ethiopia and other African nations [45, 48, 65–69].

Mothers attended a primary education, the likelihood of their children receiving the full pentavalent vaccination was higher. The current study report is supported by study findings of Arbaminch, Jigjiga, Southwest Ethiopia, Eretria, Togo, Kenya, Ghana, Zimbabwe, and Burkina Faso [42, 44, 49, 69–80].

Children who belonged to the wealthy family had a higher chance of receiving the full pentavalent vaccination than children from poverty-stricken families. The current study report is in line with the studies done in Ethiopia [23, 80], sub-Saharan Africa [36], Nigeria [34], Togo [41], Maynamar [81], Malawi [82], and Democratic Republic of Congo [83]. The possible justification could be wealthier families tend to adopt healthier childcare practices and demonstrate better health-seeking behaviors. In contrast, the travel costs to healthcare facilities may discourage poorer families from vaccinating their children [40].

Those mothers who gave birth in health institutions a had higher chance of their children receiving the full pentavalent vaccination compared to home deliveries. This is comparable to findings in Ethiopia [84], Kenya [85], the Philippines [86], and Pakistan [87]. This shows that increased contact with the healthcare facility would improve full pentavalent vaccination [17, 24, 41, 49, 71, 88–93]. The fact that mothers give birth in health institutions are more likely to have access to healthcare related information and receive advice about vaccination can explain this finding. The birth order of the children had been identified as the determinants of full pentavalent vaccine uptake. In children whose birth order was between one and three, the odds of receiving full pentavalent vaccination were higher compared to children whose birth order was six or more. This is in agreement with various former study findings as family size grows, resources like time and attention are divided among children, which may lead to late-born children not receiving the complete vaccine series [68, 94, 95].

Despite generating this essential evidence, maternal verbal response was among the modalities used to collect data on basic vaccinations; This may introduce recall bias, and potentially leading to either an underestimation or overestimation of vaccine uptake.

## Conclusion

In Ethiopia, the total uptake of full pentavalent vaccination was very low compared to the world’s target. Spatial cluster analysis revealed that most of low coverage regions for full pentavalent vaccine uptake were Afar, Somali, and Harari. Higher maternal age, maternal primary education attended, rich wealth index, 1–3 birth order, and giving birth in a health care setup were the determinants of full pentavalent vaccine uptake. Special attention should be given to Afar, Somali, and Harari regions, to strengthen the vaccine uptake. Moreover, improved socioeconomic status and getting maternal health services during delivery are necessary to enhance vaccine uptake.

## Supporting information

S1 Data SPSS version dataset. (SAV)

## Abbreviations

AOR: Adjusted odds ratio
DPT: Diphtheria, pertussis, and tetanus
EA: Enumeration area
EDHS: Ethiopia Demography, Health Survey
EPI: Expanded program of immunization
SNNP: South Nation’s Nationalities and Peoples
VPD: Vaccine preventable disease
WHO: World Health Organization
